# *Citrus tristeza virus* P33 Protein Is Required for Efficient Transmission by the Aphid *Aphis* (*Toxoptera*) *citricidus* (Kirkaldy)

**DOI:** 10.3390/v12101131

**Published:** 2020-10-06

**Authors:** Turksen Shilts, Choaa El-Mohtar, William O. Dawson, Nabil Killiny

**Affiliations:** Plant Pathology Department, CREC-IFAS, University of Florida, 700 Experiment Station Road, Lake Alfred, FL 33850, USA; tkshilts@ufl.edu (T.S.); mohtarc@ufl.edu (C.E.-M.); wodtmv@ufl.edu (W.O.D.)

**Keywords:** *Citrus tristeza virus*, aphid transmission, virus–vector interaction, P33 protein, virus recombinant, *Aphis* (*Toxoptera*) *citricidus* (Kirkaldy)

## Abstract

Plant viruses are threatening many valuable crops, and *Citrus tristeza virus* (CTV) is considered one of the most economically important plant viruses. CTV has destroyed millions of citrus trees in many regions of the world. Consequently, understanding of the transmission mechanism of CTV by its main vector, the brown citrus aphid, *Aphis* (*Toxoptera*) *citricidus* (Kirkaldy)*,* may lead to better control strategies for CTV. The objective of this study was to understand the CTV–vector relationship by exploring the influence of viral genetic diversity on virus transmission. We built several infectious clones with different 5′-proximal ends from different CTV strains and assessed their transmission by the brown citrus aphid. Replacement of the 5′- end of the T36 isolate with that of the T30 strain (poorly transmitted) did not increase the transmission rate of T36, whereas replacement with that of the T68-1 isolate (highly transmitted) increased the transmission rate of T36 from 1.5 to 23%. Finally, substitution of *p33* gene of the T36 strain with that of T68 increased the transmission rate from 1.5% to 17.8%. Although the underlying mechanisms that regulate the CTV transmission process by aphids have been explored in many ways, the roles of specific viral proteins are still not explicit. Our findings will improve our understanding of the transmission mechanisms of CTV by its aphid vector and may lead to the development of control strategies that interfere with its transmission by vector.

## 1. Introduction

Hundreds of plant pathogens are transmitted by insect vectors. The order Hemiptera contains the most common insect vectors (aphids, leafhoppers, psyllids, and whiteflies), which are responsible for the transmission of many destructive plant diseases [1]. Plant viruses threaten many economically important crops, and the *Citrus tristeza virus* (CTV) is considered one of the most economically important plant viruses [2]. CTV is a member of the *Closteroviridae* family, and it is transmitted by several aphid species, including the cotton or melon aphid (*Aphis gossypii* Glover), the spirea aphid (*Aphis spiraecola* (Patch)), the black citrus aphid (*Toxoptera aurantii*), and the brown citrus aphid (*Aphis* (*Toxoptera*) *citricidus*) [3].

CTV is one of the most ubiquitous viruses infecting citrus, and it has destroyed more than 100 million citrus trees that were grafted on sour orange (*Citrus aurantium*) in the United States, Brazil, Argentina, Venezuela, and Spain [4]. The symptoms of the disease caused by CTV depend on the citrus variety, CTV strain, and the selected rootstock [5]. Stem pitting is observed in infected sweet orange, mandarin, and grapefruit, whereas lemon and sour orange show stunting and yellowing [5]. In general, plants grafted on sour orange are very sensitive to CTV and show quick decline and dieback.

The brown citrus aphid, *A. citricidus,* is found in most of the citrus-growing areas of the world and is considered as the most efficient vector for the CTV strains [6]. The brown citrus aphid host range is limited to mainly rutaceous species and transmits most of CTV isolates more efficiently than other aphids [7]. The brown citrus aphid transmits the CTV in a semipersistent manner; it requires from a few seconds to 30 min to acquire the virus and several hours to inoculate it into a new host plant [8]. CTV-infected *A. citricidus* can retain the virus for 24–48 h [8].

The transmission efficiency of CTV by aphids depends on multiple factors, including the aphid species and CTV genotype [9]. CTV consists of at least six different strains that exhibit a variety of different phenotypic characteristics. T30, T68, T36, T3, VT, and RB are the most common strains of CTV, sharing an average of 80.5–92.4% nucleotide identity across the length of the CTV genome [10]. It was also noted that the 5′-end of the genome is more diverse than the 3′-end of the genome [11]. Transmission rates of CTV by aphids are varied among strains. The transmission rate of the T36 strain by brown citrus aphids is less than 2% [3,12], while the transmission efficiency of NZ-B18 (natural recombinant of T68 and VT) by brown citrus aphids is more than 40% transmission [13]. In addition, the interaction between CTV genotypes affects the transmission rate depending on the coinfection of different genotypes [9]. Previous studies showed that individual aphids can acquire and transmit only some of the genotypes present in the source plant [9].

The molecular mechanism of CTV aphid transmission is not fully understood. According to Ng and Falk [14], the transmission occurs by direct interaction between the virion coat proteins or the nonvirion helper component. They based their conclusion on the fact that CTV is not aphid-transmissible as ultra-purified virions fed to aphids in vitro [15]. They concluded that transmission did not occur because of the absence of the required helper protein; however, this experiment has not been replicated [14]. On the other hand, sucrose gradient extraction (partial purification) is used for CTV virions extraction regularly and is successful for the experimental CTV inoculation of citrus [16]. However, it should be considered that the extraction of intact particles is difficult, and it has been shown that partially unencapsidated or fragmented CTV is less infectious (10^4^–10^5^ times) than intact virions [17].

CTV encapsidates into long, flexuous virions, which occur via a complex mechanism. The polar virions are composed of two capsid proteins and a single-stranded, positive-sense genomic RNA (gRNA) of approximately 20 kb, containing twelve open reading frames (ORFs) and two untranslated regions (UTRs) [18]. The 5′-half of the genome encompasses two ORFs encoding proteins associated with viral replication that are expressed from the genomic RNA called the *replication gene blocks* and involve ORF 1a (encodes a 349 kDa polyprotein) and 1b (encodes a 54 kDa protein—RdRp domains) [18]. The ten 3′-proximal ORFs are expressed via 3′-coterminal subgenomic RNAs, and these include genes for the minor coat protein (CPm of 27 kDa—P27) and major coat proteins (CP 25 kDa—P25) and proteins P33, P6, P65, P61, P18, P13, P20, and P23 [18]. The CP, CPm, P61, and P65 (HSP70h) are essential for virus particle assembly, and P61 is involved in the systemic invasion of the host plant [19]. The CTV HSP70h (65 kDa) is a homolog of the HSP70 plant heat-shock proteins [18]. The P20 protein is a homolog of P21 of beet yellows closterovirus (BYV), whereas P33, P18, P13, and P3 have no homologs in the other closteroviruses [20]. CTV proteins P20, P23, and CP are involved in the suppression of RNA silencing [21], whereas the P33, P18, and P13 proteins are CTV host-range determinants, with P33 being of particular interest due to its multifunctional nature and possible role in viral movement [16,22].

In recent work, the effects of mutation and variation in viral genes on CTV transmission by the brown citrus aphid was investigated by exchanging *p65* (Hsp70) and *p61* from the highly aphid-transmissible CTV isolate FS577 (24.1%) into the poorly transmissible CTV strain, T36 clone [3]. Replacement of both genes (*p65* and *p61*) increased the transmission rate of T36 from 0.6 to 18%, while substitution of *p61* or *p65* individually increased the transmission only to 4% and 2%, respectively [3]. This finding suggested that the transmission process requires the cooperation of *p65* and *p61* genes, which are required for virion assembly [3]. Using fluorescently labeled virions, Killiny et al. [23] showed that CTV binds specifically to the lining of the cibarium of the aphid. In addition, in vitro competitive binding assays between fluorescent virions and free viral proteins showed that CPm is involved in virus–vector interaction, and that CPm (P27) and HSPs (P61 and P65) were also implicated in CTV binding to the cibarium [23]. Furthermore, it was reported that the CPm is involved in vector interaction in other characterized closteroviruses [14]. To date, it is not very clear which CTV genes or sequences are related to aphid transmission, and no work has been done to determine whether the replication-associated proteins are involved in aphid transmission.

The focus of this study is to understand the CTV–vector relationship by exploring the influence of viral genetic diversity on virus transmission. We built several infectious clones with different 5′-proximal ends from different CTV strains and assessed their transmission by the brown citrus aphid. To construct the new clones, we included a sequence from the replication gene block and *p33* from the T30 and T68 isolates. Subsequently, we examined the viral determinants that affect rates of aphid transmission by examining chimeric viruses created by the substitution of sequences from the highly transmitted T68 isolate into the poorly transmitted T36 isolate. The results of this study will give insights about the effects of genetic sequence variations of CTV strains on the CTV–vector transmission.

## 2. Materials and Methods

### 2.1. Citrus Plants, CTV Isolates, and Aphid Colonies

The CTV isolates T30, and T68-1, the infectious clone T36, and the series of hybrids engineered in this work (35s200, 35s230-37, 35T8) were maintained in one-year-old *C. macrophylla* plants under controlled greenhouse conditions (23 ± 1 °C, 65 ± 5% RH (relative humidity), and a photoperiod of 16/8 h (day/night)). These plants were roughly two feet tall, with a single stem, and used as a source of virus for later graft inoculations of CTV seedlings. The brown citrus aphid (*A. citricidus)* was used for the transmission of CTV. Healthy *A. citricidus* were reared on uninfected healthy *Choisya ternata* plants in insect-proof cages (61 × 61 × 92 cm). Aphid colonies and citrus plants were located at the Citrus Research and Education Center, UF-IFAS, Lake Alfred, FL.

### 2.2. Construction of CTV Hybrids

Three CTV hybrid infectious clones were constructed by using the CTV-T36 full-length (GenBank accession # AY170468) infectious clone plasmid [24,25] as a cloning vector backbone. Fragments produced by reverse transcription PCR (RT-PCR) from T30 (GenBank accession #AF260651.1) [26], and the T68 isolates (GenBank accession #JQ965169.1) of CTV were used for substitution into the CTV-T36 cloning vector. A list of primers used in the construction of the CTV infectious clones is shown in Table 1. 

Initially, we localized the unique restriction sites (ApaI, AscI, PspXI, Bsu36I, XmaI, and PmeI) within the genome of CTV strains to be able to exchange fragments between the different strains. After the construction of CTV chimeric sequences, the new constructs were confirmed by Sanger sequencing (Macrogen, Rockville, MD, USA).

### 2.3. Reverse Transcription PCR (RT-PCR)

Total RNA extraction was performed using Trizol according to the manufacturer’s recommendation. The AffinityScript One-Step RT-PCR Kit (Bio-Rad, Hercules, CA) was used as a complete system for single-tube RT-PCR to create the fragments that were used for the replacement in the CTV-T36 infectious clone. The complementary DNA (cDNA) was amplified in the same tube by Herculase II fusion DNA polymerase. Complementary DNA synthesis and PCR took place during an uninterrupted thermal-cycling program. cDNA synthesis occurred at 50 °C for 50 min immediately followed by an RT heat inactivation step at 94 °C and then 40 thermal cycles for PCR amplification. The amplification cycle consisted of a denaturation step at 94 °C for 30 s, a template primer annealing step at 58 °C for 30 s, and an extension step at 68 °C for 1 min if the target was bigger than 1 kb, or 72 °C for targets smaller than 1 kb.

### 2.4. CTV T36/T30 Infectious Hybrid Clone (35s230-37)

**Figure 1:**
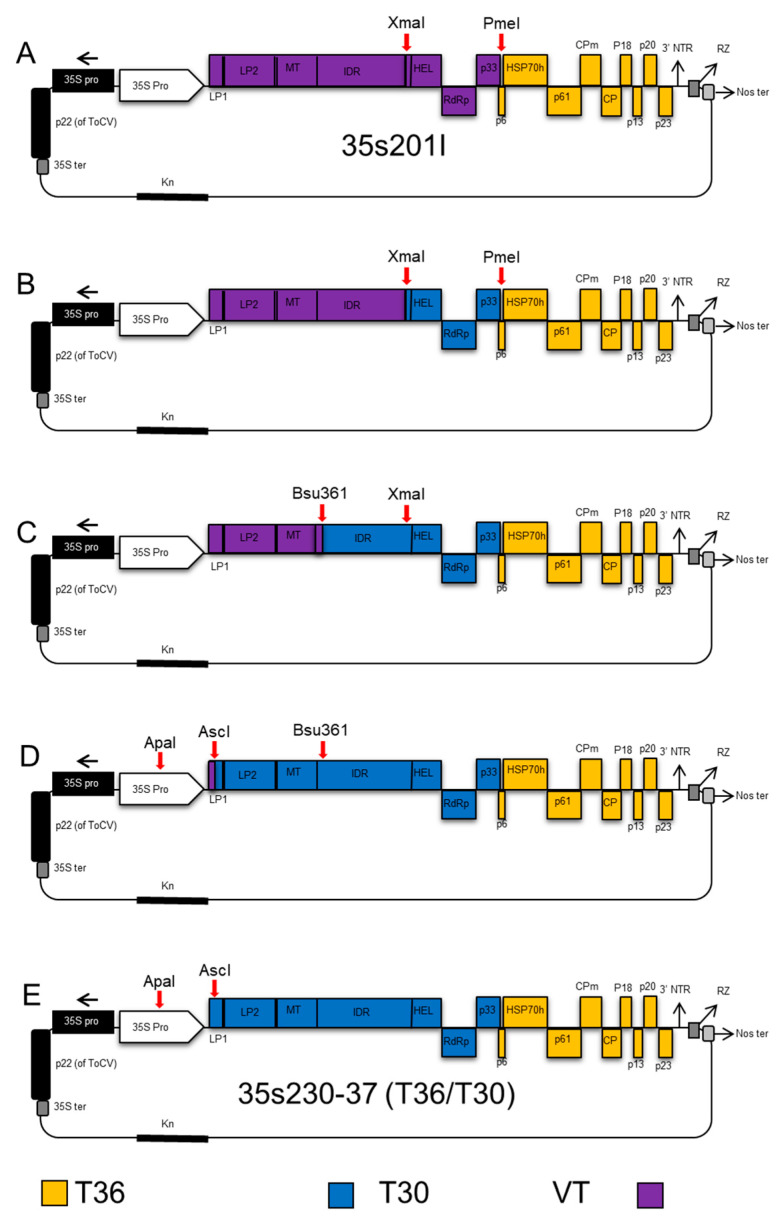
Schematic representation describing the cloning steps of the *Citrus tristeza virus* (CTV) T30-T36 (35s230-37) infectious hybrid clone. (**A**) The 35s201I plasmid vector of VT-T36 was used as a backbone clone; (**B**) the fragment containing the helicase, *RdRP*, and *p33* was amplified from strain T30, and ligated into plasmid vector 35s201I, which was digested with restricted enzymes XmaI and PmeI; (**C**) the fragment containing leader protease LP2 to helicase from T30 was amplified and ligated into the plasmid vector after digesting with Bsu361 and XmaI; (**D**) the fragment covering nucleotides 182 to 4431 from the T30 strain was amplified and ligated into the vector after digestion with AscI and Bsu36I restriction enzymes; (**E**) the fragment containing the AscI restriction enzyme site until the 182 nt was amplified and added to the plasmid vector digested with ApaI and AscI restriction enzymes to produce the new infectious hybrid clone named 35s230-37 used for studying the transmission of CTV.

The T30 infectious hybrid clone was constructed in four ligation steps through replacement of four fragments of the T30 isolate and exchanging fragments into an infectious clone of T36-based vector 35s201I, which is a chimeric hybrid of a VT-T36 infectious cDNA clone from our lab inventory (Figure 1A), by swapping from the 3′-end toward the 5′-end. First, an amplified cDNA fragment comprised of nucleotides 8083 to 11828 was amplified using forward primer c2459 and reverse primer c2281.

The PCR product was digested with PmeI and XmaI and ligated into the CTV-T36 infectious plasmid vector 35s201I digested with the same restriction enzymes (Figure 1B). The second fragment between nucleotides 4431 and 8083 using forward (c2356) and reverse (c2358) was digested with Bsu36I and XmaI and then ligated into the similarly digested CTV hybrid clone from the previous step (Figure 1C). Then, the PCR product amplified between nucleotides 182 and 4431 using forward (c2268) and reverse (c2357) was digested by AscI and Bsu36I, and ligated into the CTV hybrid clone from the second step, which was digested with the same restriction enzymes (Figure 1D). The final CTV hybrid clone was constructed by inserting an amplified cDNA fragment produced by RT-PCR using forward (c2267) and forward (c1882) primers. The PCR product amplified between the AscI restriction enzyme site in the nucleotide ApaI restriction site in the 35s promoter of CaMV (c1882). The previous hybrid clone from the third step was digested with ApaI and AscI restriction enzymes and was ligated with the amplified fragment. The final infectious clone containing T30 ORF1a and 1b in addition to *p33,* and the intergenic region upstream of *p6* was named 35s230-37 (Figure 1E).

### 2.5. CTV T36/T68 Infectious Hybrid Clone (35s200)

**Figure 2:**
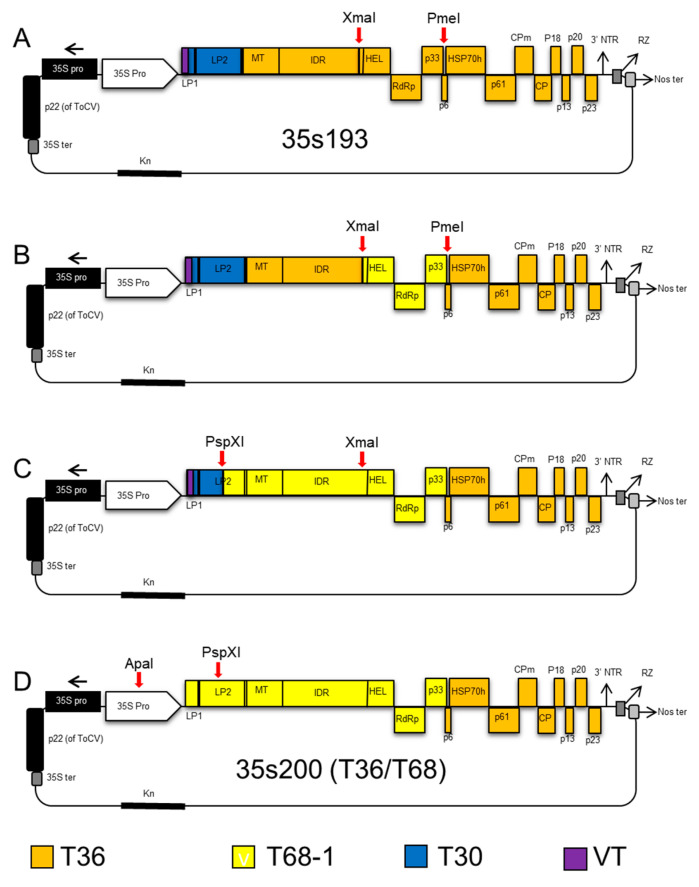
Schematic representation of cloning steps of the CTV T36-T68 (35s200) infectious hybrid clone. (**A**) The 35s193 plasmid vector of VT-T36 which was used as a backbone clone; (**B**) the fragment containing helicase, *RdRP*, and *p33* was amplified from isolate T68-1 and ligated into the CTV-T36 infectious plasmid vector 35s193, which was digested with the same restriction enzymes (XmaI and PmeI); (**C**) the fragment between leader protease LP2 and helicase from T68-1 was amplified and ligated into the plasmid vector after digesting with PspXI and XmaI; and (**D**) the fragment flanking the ApaI restriction enzyme site at the 35s (CaMV) promoter and a PspXI restriction enzyme site at nucleotide 2068 was added to the plasmid vector to produce the new infectious hybrid clone named 35s200 used for studying the transmission of CTV.

We started with CTV-T36 infectious plasmid vector 35s193 (containing the VT 5′-end sequence plus downstream sequences up to the AscI restriction enzyme site (nt 182) and L1 and L2 leader proteases of T30) as a backbone clone (Figure 2A).

The T36 infectious hybrid clone (35s200) was constructed by replacement of three fragments of the T68-1 isolate, produced by one-step RT-PCR into the T36 infectious clone. First, helicase, RdRp, and *p33* genes of the T68 strain of CTV (8078nt to 11827nt) were amplified with forward (c2299), and reverse (c2302). After digesting with XmaI and PmeI, the PCR product was ligated into similarly digested CTV-T36 infectious plasmid vector 35s193 (Figure 2B). The second step was to replace the region between leader protease LP2 and helicase. This region between nucleotides 2026 and 8078 was amplified using forward (c2296) and reverse (c2300) restriction enzymes and ligated into a similarly digested CTV hybrid clone obtained from the first step (Figure 2C). Finally, a cDNA fragment was amplified with forward (c2267) and reverse (c2298), with the PspXI restriction enzyme site at nucleotide 2068. The amplified fragment and the hybrid clone from the second step were digested with ApaI and PspXI and then ligated. This final vector including T68 ORF1a and 1b was named 35s200 (Figure 2D).

### 2.6. CTV T36 Containing P33 from the T68 Infectious Hybrid Clone (35sT8; T36/T68-P33)

**Figure 3:**
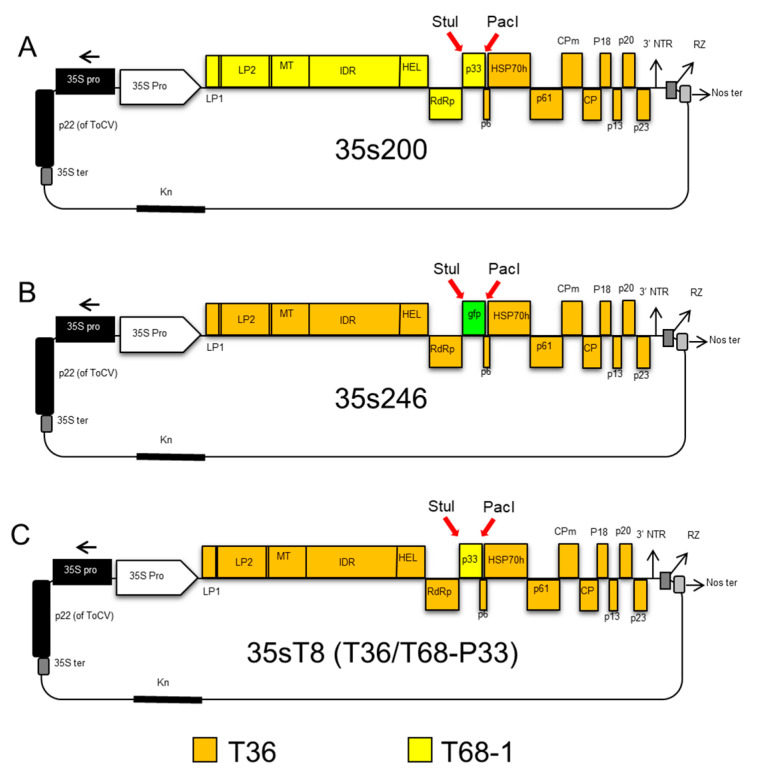
Schematic representation of cloning steps of the 35sT8 infectious hybrid created to isolate the *p33* gene from the T68 strain. (**A**) The 35s200 hybrid used for amplification of the *p33* gene of T68. (**B**) Hybrid 35s246 is an infectious cDNA clone of the T36 strain of CTV containing a *gfp* replacement of the *p33* gene. (**C**) The 35s246 hybrid was digested with restriction enzymes StuI and PacI, and the *gfp* was replaced with the amplified *p33* gene from 35s200 to produce the new infectious hybrid clone named 35sT8.

To determine if the P33 protein is solely responsible for aphid transmission of CTV, we used the 35s200 infectious cDNA clone as a template to amplify *p33* gene of T68 strain (Figure 3A), using forward primer c2475 containing a StuI restriction site and reverse primer c2470 having a PacI restriction enzyme site.

The PCR product was used for replacement between the StuI–PacI restriction enzyme sites in the infectious CTV clone 35s246 that has the *gfp* at this position (from our lab inventory) (Figure 3B). This CTV-T68/T36 infectious hybrid clone was named 35sT8, and it included only the P33 gene from the T68 isolate of CTV (Figure 3C).

### 2.7. Agrobacterium Infiltration of CTV Constructs into Nicotiana Benthamiana

The cell line of *Agrobacterium tumefaciens* strain EHA 105 was used for the transformation of CTV constructs produced in this study. *Agrobacterium*-mediated inoculation of CTV constructs into *N. benthamiana* was performed as previously described, with minor modifications [27]. To promote the CTV infection in *N. benthamiana*, different silencing suppressors, including *p22* [28], *p19* [27], *p24* [29] and P1/HC-Pro [30], were transformed into the EHA 105 cell line and coinfiltrated with the CTV clones [31,32].

### 2.8. CTV Virion Isolation and Bark Flap Inoculation into Citrus

After 8–9 weeks post-infiltration into *N. benthamiana*, the systemic infection of CTV in *N. benthamiana* leaves was confirmed using ELISA [33,34]. CTV virion isolations for bark flap inoculation of *C. macrophylla* were performed as previously described [27,35] with an additional centrifugation step. Seedlings 12–18 months old and approximately 24” in height with stems of pencil thickness were selected for bark flap inoculation [35]. Citrus plants were grown under the greenhouse conditions described earlier.

### 2.9. Enzyme-Linked Immunosorbent Assay (ELISA)

Double antibody sandwich (DAS) ELISA was performed using rabbit polyclonal antibodies as described previously [33]. A new flush of citrus plants or young bark tissue was collected (around 0.25 g). Extracts were prepared by homogenizing the tissue in 5.0 mL of phosphate-buffered saline with a pH of 7.8, containing 1% Tween-20 (PBS-Tween). Rabbit polyclonal antibody (1 μg/mL) was used for coating the ELISA plate. The CTV detection antibody and enzyme conjugate (A + B) was used at 1:750K dilution (Agdia, Elkhart, IN, USA). Samples were replicated twice per plate. Reactions were read spectrophotometrically at 405 nm and filtered on an ELx800 Universal Microplate Plate Reader (Bio-Tek Instrument Inc., Winooski, VT, USA) after 30 and 60 min.

### 2.10. Aphid Transmission Assays

The apterous form of *A. citricidus* was used for the transmission studies. Aphids were maintained on virus-free *Choisya ternata* plants, then fed on CTV graft-inoculated *C. macrophylla* for a virus-acquisition access period of 24 h. Groups of aphids were placed on virus-free Mexican lime (*C. aurantiifolia*) seedlings (5–7 aphid/plant). Replicates were up to 300 per treatment. The aphids were placed on the seedlings for an inoculation access period of 24 h. After feeding, the plants were sprayed with aphicide (Malathion) to kill the aphids. Then, the plants were transferred to the insect-free greenhouse and were maintained at ambient temperatures for up to four months. Plants were checked for transmission of CTV by ELISA at eight weeks after inoculation. The transmission efficiency was defined as the percentage of infected plants. The proportions of infected to healthy plants were compared among the treatments, using a one-by-six contingency table analysis.

## 3. Results

### 3.1. The Constructed Infectious Hybrid Clones Replicate Normally in C. macrophylla

As many genes are involved in replication and virion assembly, it was necessary to ensure that changes in the genome of T36 infectious clone did not affect virus replication within the plant. For that reason, donor plants used for aphid transmission experiments (5 plants/treatment) were tested for CTV by ELISA. Table 2 shows that similar ELISA results were obtained for plants used in CTV transmission experiments. Therefore, we excluded the possibility of a direct correlation between the aphid transmission rate and the level of virus titer in donor plants.

### 3.2. Replacement of 5′-End Terminus Genes (lp1 to p33) of the Poorly Transmitted Isolate T36 with those from the Highly Transmissible Isolate T68-1 Significantly Increases the Transmission Rate by A. citricidus

**Figure 4:**
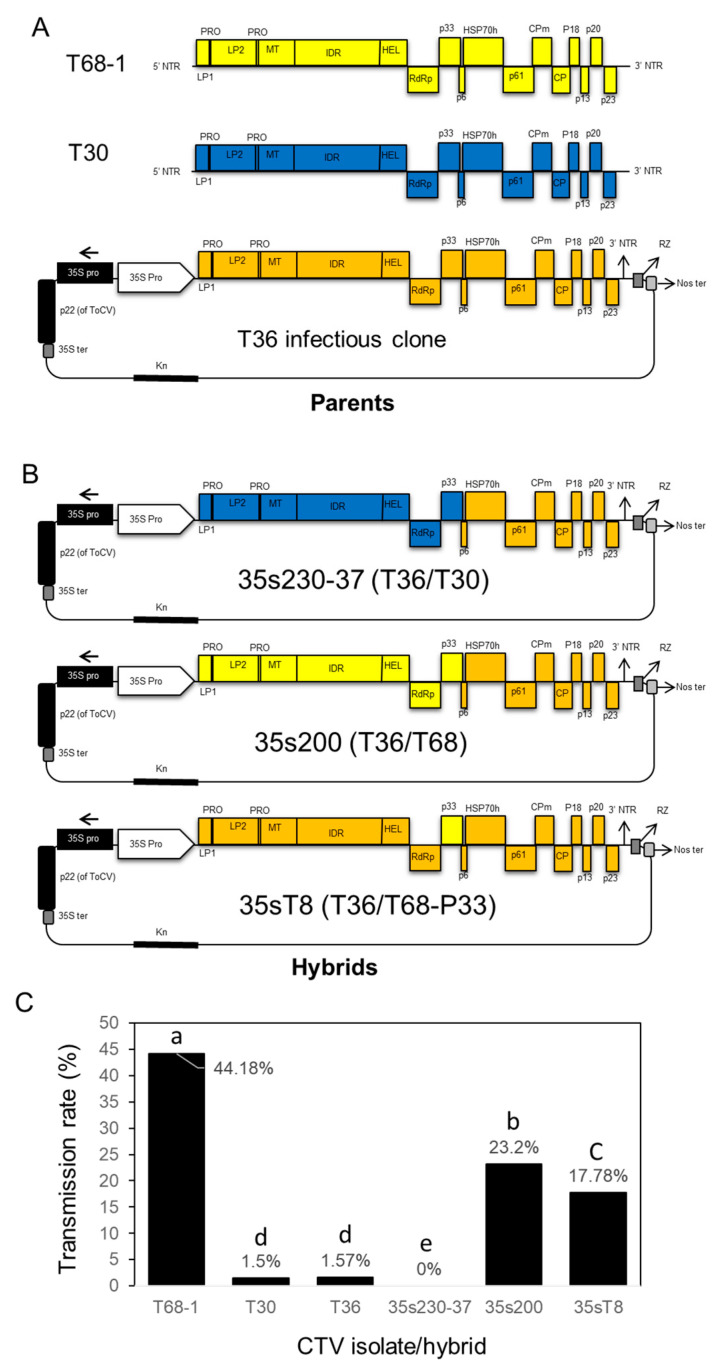
Aphid transmission of CTV strains and constructed hybrids. (**A**) Schematic representation of the genome of the parent CTV isolates (T68-1, T30, and T36 infectious clone) used to construct the hybrids. (**B**) Schematic representation of the infectious hybrid clones (35s230-37, 35s200, and 35sT8) showing the replaced genes. (**C**) Transmission rates of the parent isolates and the constructed infectious hybrids by *A. citricidus.* Genes from T36 are colored in orange, genes from T68-1 are colored in blue, and genes from T30 are colored in yellow. Treatments with different letters on bars indicate statistically different transmission rates (*p* < 0.05).

The parent isolates (T68-1 and T30) and the infectious clone (T36) were tested for their transmissibility by *A. citricidus* to provide a baseline for the hybrids constructed in this study (Figure 4A).

In order to examine the effect of genetic variation of CTV strains, and particularly the 5′-end terminus on vector transmissibility, we constructed two CTV hybrids by replacement of the 5′-endof CTV-T36 (poorly transmitted infectious clone) with the 5′-end of CTV-T30 (another poorly transmitted infectious clone) or with that of the highly transmitted strain CTV-T68 (Figure 4B). The transmission rate of CTV parent isolates, including T30, T68-1, and the infectious clone T36, are presented in Figure 4C. The aphid transmission experiments were completed using a total number of 215 plants for T68-1 with three replications, 127 plants for T30 with four replications, and 66 plants for T36 with three replications. The transmission rates were 44.18% (95 positives from 215 transmissions), 1.57% (2 positives from 127 transmissions), and 1.5% (1 positive from 66 transmissions) for T68-1, T30, and T36 isolates, respectively. These findings confirmed our knowledge that T30 and T36 isolates are poorly transmitted by *A. citricidus,* while the T68-1 isolate is highly transmitted by the same vector. The replacement of the 5′-end terminus of the T30 isolate in the T36 infectious clone did not increase the transmission rate compared to the original isolates (T36 and T30). While the T36/T30 hybrid clone had a 0% transmission rate, the hybrid of T36/T68 was transmitted with a rate of 23.20% (71 positives from 306 transmissions), indicating that the 5′-end terminus of T68 contains the transmission determinant(s) for the transmission rate of CTV-T68, which was the highest.

### 3.3. Replacement of p33 from CTV-T36 with p33 from CTV-T68-1 Significantly Increased the Transmission Rate by A. citricidus

The aphid transmission experiment was replicated four times to determine if the P33 protein is required for aphid transmission of CTV. The accumulative results showed the 35sT8 hybrid (T36/T68-P33) was transmitted with a frequency of 17.78% (16 positives out of 90) (Figure 4C), suggesting that successful aphid transmission requires the P33 protein and that the poorly transmitted strains may have a mutated *p33* gene.

## 4. Discussion

Vector transmission of plant viruses is an important area of research in plant pathology. Before the era of genome sequencing and bioinformatics analysis, basic vector transmission information was included as a criterion in plant virus taxonomy. Although interactions between viruses and vectors are very specific and unique to each virus, the vector transmission has a common basis for plant viruses. Plant viruses are retained in the vector until they are transmitted to their plant hosts. The virion binding sites are very precise and bind only to specific sites of the mouthparts or foregut in vectors [36,37]. To date, the molecular mechanisms of CTV transmission by aphid species are not fully understood, even though they have been addressed in several studies.

Our results showed that the transmission rate by brown citrus aphids of the T36 and the T30 isolates was very low (<2%), whereas the transmission rate of T68 was very high (44%). These results are in agreement with previous reports [3,10,12,13]. Taken together, these results indicated that the T68 genome contains a specific gene or genes that could be involved in CTV transmission. In harmony with this suggestion, we found that replacement of the 5′- end of the T36 isolate with that of the T68-1 isolate increased the transmission rate of T36 from 1.5 to 23%. In addition, replacement of the 5′- end of the T36 strain with that of the T30 strain did not affect the transmission rate of T36. Our results indicated that the 5′- end of the highly transmissible T68-1 isolate contains a gene or genes that is/are involved in CTV transmission by the brown citrus aphid.

The exchange of the 5′- end of the T36 strain with that of T68-1 isolate could affect the new hybrid assembly and replication. Therefore, to determine whether differences in transmission efficiency were caused by an increase in the hybrid’s replication (titer) in the source plants, we tested the CTV titer in the donor plants used for aphid transmission assays by ELISA. The ELISA assays showed that the source plants (*C. macrophylla*) accumulated similar titer from the different virus isolates and hybrids, indicating that substituting the 5′-end of the genes did not affect virion assembly or replication in the graft-transmitted donor plants. These results demonstrated that the increased transmission rate of T68/T36 hybrid was not caused by an increase in the virus titer (replication speed).

Several studies have been conducted to elucidate the molecular mechanism of the CTV transmission by the brown citrus aphid. A previous study showed that exchanging *p65* and *p61* genes from the high aphid-transmitted CTV isolate, FS577, into the infectious clone of the poorly transmitted isolate, T36, significantly increased its transmission rate [3]. Replacement of both genes increased the transmission efficiency more than the replacement of each gene, indicating that the transmission process requires the cooperation of both genes [3]. Interestingly, the previous study using competitive binding assays also showed that the heat shock-like protein (P61), P65 and the minor coat protein (CPm-P27) were implicated in CTV binding to the cibarium of the brown citrus aphid [23]. The minor coat protein (CPm) was also found to be involved in the interaction between other closteroviruses and their vectors [14].

Since our results indicated that the 5′-proximal end of the T68-1 isolate was implicated in the transmission of CTV by the brown citrus aphid, we decided to test whether the *p33* gene is solely responsible for the transmission by the brown citrus aphid. Substituting the *p33* gene from the T68 strain into the T36 infectious clone increased the transmission rate of T36 from 1.5% to 17.8%. We chose P33 because it performs multiple functions in the virus infection cycle. The *p33* gene is involved in virus movement within plants [16]. In addition, P33 was found to co-localize with the P6 protein, a putative movement protein of CTV. The previous observations suggested that P33 plays a role as a viral movement protein, which is involved in virus translocation with specific hosts [38].

A previous study also showed that the *p33* gene was required for systemic CTV infection of sour orange and lemon trees [22]. In addition, the presence of *p33* or the *p13* gene resulted in systemic infection of calamondin, and the presence of *p33* or *p18* was sufficient for systemic infection of grapefruit [22]. Interestingly, none of these non-conserved genes were required for systemic infection in certain susceptible citrus trees such as *C. macrophylla* and Mexican lime [16]. Together, these findings suggested that these three genes (*p33*, *p18*, and *p13*) extended the host range of CTV during evolution [22].

Recently, Sun and Folimonova [39] showed that the expression of *p33* gene of CTV enhanced the accumulation of reactive oxygen species (ROS) in *N. benthamiana*. On the other hand, the deletion of the *p33* gene from the CTV variant significantly decreased the ROS and programmed cell death (PCD) in *N. benthamiana* [39]. It is suggested that the P33 protein is an effector, and its presence in CTV allows *N. benthamiana* to recognize the virus and activate its immune response, which restricts CTV into the phloem tissues and decreases disease symptoms [39].

Recently, Dao et al. [40] showed that the P33 protein interacts with three viral proteins (CP, P20, and P23) in vivo and in planta. Co-expression of *p33* resulted in a shift localization of the P23 and P20 proteins toward the subcellular crude membrane fraction [40]. These four proteins co-localized in the CTV replication factories upon CTV infection [40]. Three of them (CP, P23, and P20) were present in the P33-formed membranous structures [40]. Because these three viral proteins interact with P33 and play a role in the mitigation of RNA silencing-based plant antiviral response, it is suggested that P33 and these proteins affect CTV infection in selective hosts [40]. These results indicated that CTV protein–protein interactions could play an important role in CTV transmission by its vector. Therefore, further research is needed to understand the possible role of CTV protein–protein interactions in CTV transmission by its vector.

In conclusion, herein, we showed that the P33 protein is one of the key components for the transmission of CTV by the brown citrus aphid. Previous research suggested that CTV requires two virally encoded components for successful aphid transmission, mainly P61 and P65 (HSP70h) [3]. On the other hand, P27 (CPm), P61, and P65 (HSP70h) were found to be able to bind to the lining of the cibarium of aphid foregut [23]. It is notable that in the current study, we used the highly transmissible strain T68 as a source for *p33,* while Harper et al. [3] used the highly transmissible strain FS577. The recipient in both cases was the infectious clone T36. Taken together, this outcome suggests that successful aphid transmission may require the *p33* gene and other 3′-end genes (such as *CPm*, or/and *p65*, or/and *p61*) as solitary or collaborative actors. However, this raises more questions: (i) does the P33 protein also bind to the aphid foregut? (ii) How do the four proteins (P27 (CPm), P61, and P65 (HSP70h) and P33) interact together for the highest efficiency of transmission? These questions need to be addressed in future investigations to enhance our understanding of the transmission mechanisms of various CTV strains by their vectors.

## Figures and Tables

**Table 1:** List of primers used in building CTV infectious hybrid clones.

Primer	Orientation	Restriction Enzyme	Sequence 5′—3′
C1882	Reverse		CCCGCACTTGCGGGGAGAAACCGTACG
C2267	Forward	* ApaI	GTT***GGGCCC***GATCTCCTTTGCCCCAGAGATCACAATGGACGACTT
C2268	Forward	-	AATTTCGATTCAAATTCACCCGTATCTCCGGAGCTCGAT
C2275	Forward	-	CTGAACGTGGGAAGATTGGGGATTTCAGTTTTCCGAGT
C2276	Reverse	-	ACTCGGAAAACTGAAATCCCCAATCTTCCCACGTTCAG
C2277	Forward	-	TCCTAGTCATCACTCGAGTGCCGCTCGTGGGCAACGTT
C2278	Forward	-	TCAGTTTTTCGCGATTTTTGTACGATTCGCGTTA
C2279	Forward	-	CCCGTCGCACGTGACATAACGTACAAGAAGATGACCAA
C2280	Reverse	-	AGACTATGCTCCGAATTAGTGAACGTCAAATCTTT
C2281	Reverse	-	ATAGCCACCGTCGAAAACGTGGTACCAAAGTCTA
C2296	Forward	-	AAGGTATAATTCGAGGAAGTCCTTCTATACGCG T
C2298	Reverse	-	ACTCGGAGGGCCAGCCGAACGACGACTAACACCG
C2299	Forward	-	GTT GAA GGC TGT GGG TTT CGA CAG GAA GTT AAC C
C2300	Reverse	-	ACAGGATACTTTAGTACAGTAGTCGGAAAAGTACTTC
C2302	Reverse	-	ACCAAAGTCTAGACCCAGAAGCACCATACCGCT
C2331	Reverse	Bsu36I	AGTT***CCTGAGG***TACGATTTCACGTATCTCTCTACG
C2332	Forward	Bsu36I	GTA***CCTCAGG***AACTCGTTTAATATAAGTTTCGC
C2348	Forward	-	TGAATGCTAAGACTTTTGAATGGACTTGGAA
C2352	Forward	-	ACGTAGGTGGTTGCCCATTATTTCATTTACGTAAGTTTCTGCTTCTACCTTTGA
C2353	Reverse	-	TCAAAGGTAGAAGCAGAAACTTACGTAAATGAAATAATGGGCAACCACCTACGT
C2354	Forward	-	ATATATGACCAAAATTTGTTGATGTGCAGGTGCGGGTCATACAGGAGTTCACGTTTGCA
C2355	Reverse	-	TGCAAACGTGAACTCCTGTATGACCCGCACCTGCACATCAACAAATTTTGGTCATATAT
C2356	Forward	Bsu36I	CATA***CCTCAGG***AACTCGTTTAATATGAGTTTCGTAGACT
C2357	Reverse	Bsu36I	GTT***CCTGAGG***TATGATCTCACGCACCTCTCCACA
C-2358 C2459	Reverse Forward	XmaI XmaI	GGA***CCCGGG***CAAAG CACCAAACCGTCGTCGCAAAACATGAACT TTGCGACGACGGTTTGGTGCTTTG***CCCGGG***TCCGAAAAAACGCGATTCGCT
C2470	Reverse	PacI	ACC***TTAATTAA***TCATATAAATATGATGGCTATCAAACCGCTCATT
C2475	Forward	StuI	AAT***AGGCCT***TGTTTGCCTTCGCGAGTGAAAACCAAGATATTAGAAG

Restriction sites within the primers are underlines. * Restriction site was added to the primer sequence.

**Table 2:** ELISA result of source plants used for aphid transmission of CTV.

Isolates/Hybrids	O.D. @ 405 nm *
Control	0.25 ± 0.10 ^a^
T36	3.41 ± 0.05 ^b^
WT-T68	3.50 ± 0.04 ^b^
WT-T30	3.02 ± 0.20 ^b^
35s230-37 (CTV T36/T30)	3.19 ± 0.20 ^b^
35s200 (CTV T36/T68)	3.40 ± 0.10 ^b^
35sT8 (CTV T36/T68-P33)	3.36 ± 0.02 ^b^

* Different letters indicate statistically significant differences (*p* < 0.05). Statistical analysis was performed using ANOVA followed by post hoc pairwise comparison (Tukey). Five plants were used for each isolate or infectious clone.
